# Activation of G-Protein-Coupled Estrogen Receptor 1 (GPER1) Reduces Progression of Vulvar Carcinoma Cells

**DOI:** 10.3390/ijms241813705

**Published:** 2023-09-05

**Authors:** Johanna Loris, Lena Hanesch, Gerd Bauerschmitz, Julia Gallwas, Carsten Gründker

**Affiliations:** Department of Gynecology and Obstetrics, University Medical Center Göttingen, 37075 Göttingen, Germany; johanna.loris@stud.uni-goettingen.de (J.L.); lena.hanesch@stud.uni-goettingen.de (L.H.); gerd.bauerschmitz@med.uni-goettingen.de (G.B.); julia.gallwas@med.uni-goettingen.de (J.G.)

**Keywords:** G-protein-coupled estrogen receptor, GPER1, vulvar carcinoma, tumor suppressor, oncogene

## Abstract

Whether G protein-coupled estrogen receptor 1 (GPER1) is tumor-promoting or tumor-suppressive depends in part on tumor entity. Little is known about the function of GPER1 in vulvar carcinoma. In this work, we aim to clarify what role GPER1 plays in vulvar cancer, tumor-promoting or tumor-suppressive. Localization of GPER1 in A431 and CAL-39 vulvar carcinoma cells was examined by immunofluorescence. Using a tissue microarray of vulvar neoplasias, the correlation between GPER1 expression and grade of malignancy was investigated. A431 and CAL-39 cells were treated either with GPER1 agonist G1 or antagonist G36. Proliferation was quantified by BrdU assay and viability examined using Resazurin assay. Morphological changes were analyzed by microscopy and measured using ImageJ. Cell migration was analyzed by gap closure assay. Clonogenic potential was tested by colony and sphere formation. Expression of estrogen receptors was examined by Western blot. GPER1 was found consistently expressed in vulvar neoplasia tissues. The immune-reactive score was found to be significantly higher in tissue samples of lymph node metastases and neoplasias with grade 3. In A431 and CAL-39 vulvar carcinoma cells, GPER1 expression was mainly found in the cytoplasm and nuclei. Treatment of A431 and CAL-39 cells with GPER1 agonist G1 resulted in a decrease in proliferation and migration. In addition, colony formation and tumor sphere formation were reduced. Furthermore, morphological signs of necrosis and reduction in cell viability after G1 treatment were observed. The GPER1 antagonist G36 did not have significant effects on vulvar carcinoma cells. Neither agonist G1 nor antagonist G36 treatment resulted in altered expression of estrogen receptors. Activation of GPER1 with GPER1 agonist G1 reduces the tumorigenic potential of the vulvar carcinoma cells. It can be deduced from this that GPER1 appears to have a tumor-suppressive effect in vulvar carcinoma.

## 1. Introduction

Vulvar carcinomas are uncommon, accounting for 3–5% of all gynecological cancers [1,2,3]. Their etiology is diverse with squamous cell carcinoma (SCC) being by far the most common subtype. Around 15–25% of SCC are induced by high-risk human papillomavirus (HPV) occurring in younger women with increasing incidence, whereas most vulvar carcinomas are HPV-negative and associated often with lichen sclerosus, primarily affecting postmenopausal women [1,4]. Vulvar cancers are regarded to be nonhormone-dependent [5]. Their treatment is predominantly surgical, although concurrent chemoradiation is an effective alternative, particularly for advanced tumors [6]. Management should be individualized and carried out by a multidisciplinary team experienced in the treatment of these tumors. When treatment options are considered, the most appropriate treatment of the primary lesion and the inguinal lymph nodes should be considered independently of each other to maximize the chance of cure while minimizing morbidity. The 5-year survival rates vary between 86% if the tumor has not spread to lymph nodes or nearby tissues and 54% in case of lymph node involvement [7]. As the diagnosing of lymph node metastasis in earlier stages has not improved since the 1980s, a reconsideration of the screening and care model is needed [8].

The G-protein-coupled estrogen receptor 1 (GPER1, GPR30) is a seven transmembrane-domain G protein-coupled receptor that is mainly located in the endoplasmic reticulum [9]. In gynecological neoplasms, the receptor is described to be located in cytoplasm und nucleus [10,11,12]. The receptor is a part of cellular pathways of angiogenesis, migration, proliferation, invasion, and apoptosis [9,13,14,15]. GPER1 is also responsible for nongenomic, membrane-initiated estrogen effects [16]. According to Girgert et al. [16], the Hippo, FOXO3a, and HOTAIR pathways depend on regulation by GPER1. Furthermore calcium signaling, cAMP, epidermal-growth-factor (EGF) receptor, and IxB pathways are influenced by GPER1 [16]. For the experiments in this study, GPER1 analogs G1 and G36 were used. G1 is a nonsteroidal compound that is a highly selective and potent agonist of GPER1 [9]. It does not bind to the estrogen receptors (ER) α and β [9,17]. G36 is a nonsteroidal, selective antagonist of GPER1, which does not bind to ERα or ERβ either. Further, it blocks activation of phosphoinositide-3-kinase and prevents activation of extracellular signal-regulated kinase by G1 or estrogen [17].

GPER1 is expressed by many hormone-sensitive tumor entities and rapidly activates signaling cascades mediated by estrogen, making it a potential target for carcinoma treatment [13]. GPER1 has been detected in tissues including the testis, ovaries, breast, endometrium, and lung [15]. GPER1 is likely to modulate carcinogenesis [14]. The tissue type seems to define the effects of the receptor [15]. In cutaneous neoplasms, the effects of estrogen signaling are largely unexplored [18,19]. Bai et al. [13] reported the function of GPER1 as an oncogene in the skin, but the treatments with estrogens and its antagonists led to conflicting results [12,18]. GPER1 has a tumor-suppressive function in ovarian carcinoma [20]. In breast carcinoma, GPER1 is often described as an oncogene [21,22,23,24,25], but Han et al. [20] observed a tumor-suppressive effect. Tumor-suppressive [14,26,27,28] and oncogenic effects [11,29,30] are discussed in cervical carcinoma. In vulvar carcinoma, Lan et al. [12] suggest a function of GPER1 as an oncogene. Due to the limited evidence concerning the importance of GPER1 in the progression of vulvar carcinoma and the significance of the receptor as a therapeutic target, especially in gynecological neoplasms, it is further investigated in this study.

Using a tissue microarray of vulvar neoplasia, the correlation between GPER1 expression and grade of malignancy was explored. GPER1 was detected in A431 and CAL-39 vulvar carcinoma cells using immune cytochemical staining. Via Western blotting, the effects of treatment with GPER1 agonist G1 and antagonist G36 on estrogen receptor expression in vulvar carcinoma cell lines A431 and CAL-39 was examined. To investigate the impact of GPER1 on proliferation and viability of vulvar carcinoma cells, the BrdU and Resazurin assays were used. Effects of G1 and G36 on cell morphology of vulvar carcinoma cells A431 and CAL-39 were observed. Gap closure assay was used to analyze the migration of vulvar carcinoma cells A431 and CAL-39 after treatment with G1 and G36. To examine the ability of the vulvar carcinoma cells A431 and CAL-39 to form colonies and tumor spheres and to migrate and proliferate after being treated with G1 and G36, the colony formation and sphere formation assays were used.

## 2. Results

### 2.1. Correlation of GPER1 Expression and Grade of Malignancy within a Tissue Microarray of Vulvar Neoplasia

**Figure 1:**
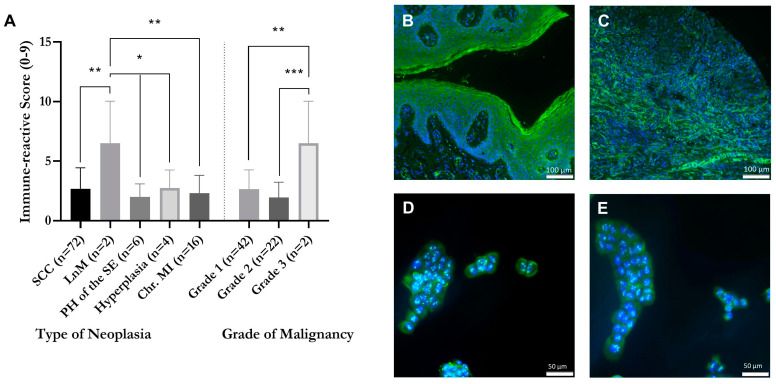
Correlation of GPER1 expression and grade of malignancy within tissue samples of vulvar neoplasia, based on FITC staining (green) of GPER1. Immune-reactive score (**A**) (0–9; staining intensity (0–3) multiplied with area size (0–3)). Detection of GPER1 epithelial in a vulva hyperplasia (**B**) and within the stroma with infiltrating tumor cells of a squamous cell carcinoma of the vulva (**C**). Magnification 10× (**B**,**C**). SCC = squamous cell carcinoma, LnM = lymph node metastasis, PH = papillary hyperplasia, Chr. MI = chronic mucosal inflammation. Mean with SEM, one-way ANOVA, *n* = 2–72. Detection of GPER1 within vulvar carcinoma cells. Cell lines A431 (**D**) and CAL-39 (**E**). Cell nuclei stained with DAPI (blue) and GPER1 stained with FITC (green). Magnification 20× (**D**,**E**). * *p* < 0.05, ** *p* < 0.01, *** *p* < 0.001.

In the evaluated microarray, GPER1 was stained green and cell nuclei were stained blue (Figure 1A–C). Each microarray was evaluated for its GPER1 stained area and its GPER1 staining intensity. The immune-reactive score gives a range of 0–9 (0 = negative, 1–3 = mild, 4–6 = moderate, 7–9 = strong) as a product of multiplication between positive-stained tumor proportion score (0–3; 0 = no positive stained tumor tissue, 1 = <30% positive stained tumor tissue, 2 = 30–60% positive stained tumor tissue, 3 = >60% positive stained tumor tissue) and staining intensity score (0–3; 0 = no color reaction, 1 = mild color reaction, 2 = moderate color reaction, 3 = intense color reaction), and then subjected to statistical analysis one-way ANOVA (Figure 1A). The immune-reactive score was found to be significantly higher in tissue samples of lymph node metastases (M = 6.5 ± 2.5; *n* = 2) compared to squamous cell carcinomas (M = 2.68 ± 0.207; *n* = 72; *p* = 0.0065), papillary hyperplasias of squamous cell carcinomas (M = 2 ± 0.447; *n* = 6; *p* = 0.0047), hyperplasias (M = 2.75 ± 0.75; *n* = 4; *p* = 0.0315), and chronic mucosal inflammations (M = 2.313 ± 0.373; *n* = 16; *p* = 0.0042). The immune-reactive score was significantly higher in neoplasias with grade 3 (M = 6.5 ± 2.5; *n* = 2) compared to neoplasias with grade 1 (M = 2.65 ± 0.22; *n* = 54; *p* = 0.0017) and grade 2 (M = 1.95 ± 0.285; *n* = 20; *p* = 0.0004). The tissue microarray was analyzed for the presence of GPER1 revealing that, among other findings, in hyperplasia an accumulation of GPER1 was observed in the epithelial layer (Figure 1B), while in squamous cell carcinoma samples an accumulation was observed in the stroma with infiltrating tumor cells (Figure 1C).

### 2.2. Detection of GPER1 in Vulvar Carcinoma Cells in Cytoplasm and Nucleus

In A431 and CAL-39, vulvar carcinoma cell GPER1 was stained green while the cell nuclei were stained blue. In both vulvar carcinoma cell lines, GPER1 was visible to the same extent in the nucleus and cytoplasm (Figure 1D,E).

### 2.3. G1 Acts through GPER1 in Vulvar Carcinoma Cells

**Figure 2:**
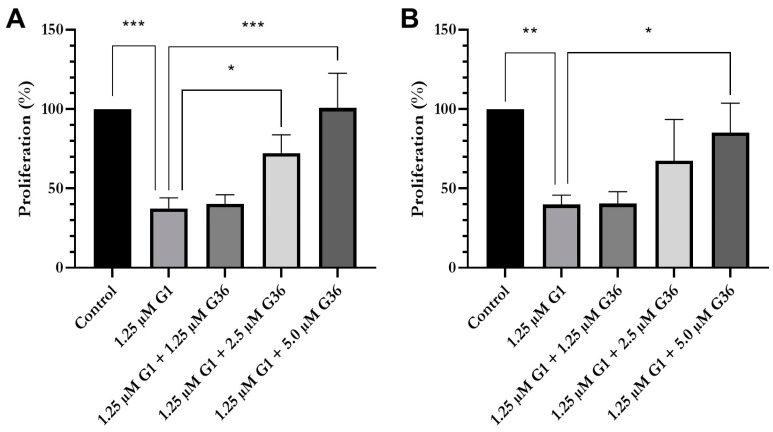
Proliferation after treatment with G1 alone and in combination with GPER1 antagonist G36. Effects of treatments with G1 or with G1 in combination with increasing concentrations of G36 on proliferation of A431 (**A**) and CAL-39 (**B**). Mean with SEM, one-way ANOVA, *n* = 3. * *p* < 0.05, ** *p* < 0.01, *** *p* < 0.001.

To show that the effect of G1 is elicited by GPER1, cells were treated with G1, both without and with increasing concentrations of GPER1 antagonist G36. The proliferation assay showed that G1 treatment (1.25 µM) significantly decreased proliferation of the vulvar carcinoma cells A431 and CAL-39 (A431: M = 37.39 ± 3.88% vs. ethanol control (= 100%); *n* = 3; *p* < 0.001 (Figure 2A); CAL-39: M = 39.91 ± 3.39% vs. ethanol control (= 100%); *n* = 3; *p* < 0.01 (Figure 2B)). This effect could be inhibited by the GPER1 antagonist G36 in a dose-dependent manner. Cotreatment of A431 cells with 1.25 µM G1 with 1.25 µM G36 resulted in a slight increase in proliferation to 40.28 ± 3.34% as compared with G1 alone (*n* = 3) (Figure 2A). After treatment with 1.25 µM G1 in combination with 2.5 µM G36, proliferation was significantly increased to 72.34 ± 6.62% vs. G1 alone (*n* = 3; *p* = 0.05). If the cells were treated with 1.25 µM G1 and 5 µM G36, proliferation was significantly increased to 100.65 ± 12.64% vs. G1 alone (*n* = 3; *p* = 0.001). After simultaneous treatment of CAL-39 cells with 1.25 µM G1 and 1.25 µM G36, the proliferation rate was 40.43 ± 4.38% (*n* = 3) (Figure 2B). After treatment with 1.25 µM G1 in combination with 2.5 µM G36, proliferation was increased to 67.44 ± 15.04% vs. G1 alone (*n* = 3). The maximum blocking effect was achieved with 5 µM G36 (M = 85.18 ± 10.71% vs. control; *n* = 3; *p* < 0.05).

### 2.4. No Impact of GPER1 Agonist G1 and Antagonist G36 Treatment on Expression of Estrogen Receptors in Vulvar Carcinoma Cells

**Figure 3:**
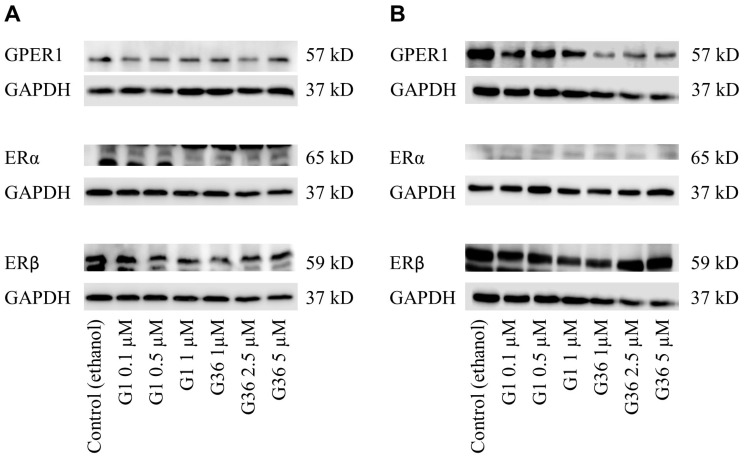
Effects of GPER1 agonist G1 and antagonist G36 on estrogen receptor expression in vulvar carcinoma cells. Expression of GPER1, ERα, and ERβ after treatment with G1 or G36 on vulvar carcinoma cells A431 (**A**) and CAL-39 (**B**). *n* = 3–4.

In the A431 vulvar carcinoma cell lines (Figure 3A), there were no changes in the expression of GPER1, ERα, and ERβ after treatment with G1 or G36, compared with control. In the CAL-39 cell line (Figure 3B), the expression of GPER1, ERα, and ERβ also remained unchanged after treatment with G1 or G36. Densiometric evaluation of 3–4 independent experiments each showed no significant effects (Figure S1, Table S1). Additional bands are nonspecific detections of the antibodies.

### 2.5. Reduced Proliferation and Viability after Treatment with GPER1 Agonist G1

**Figure 4:**
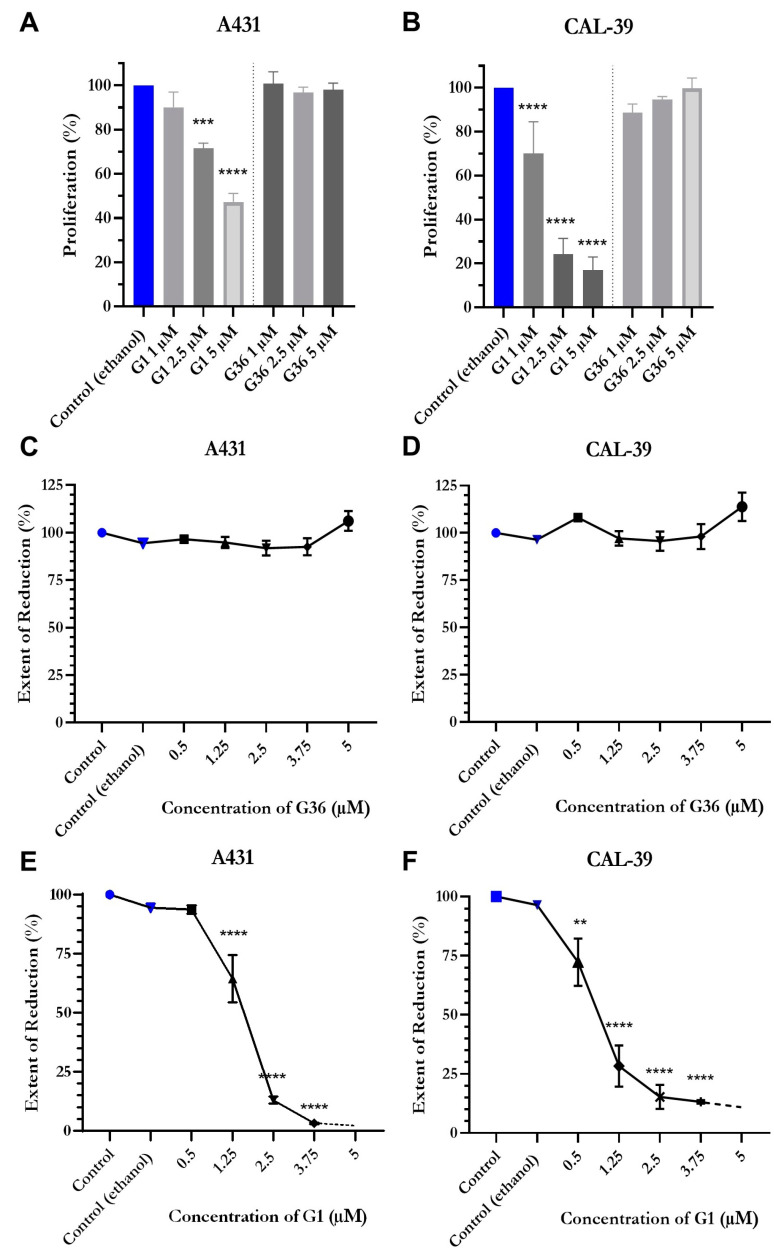
Proliferation and viability after treatment with GPER1 agonist G1. Proliferation after treatment of the vulva carcinoma cells A431 (**A**) and CAL-39 (**B**) with G1 or G36 (controls = blue). Effects of treatments with G1 or G36 on viability of A431 (**C**,**E**) and CAL-39 (**D**,**F**). Mean with SEM (**A**–**F**), one-way ANOVA (**A**–**F**), *n* = 3 (**A**,**B**), *n* = 5–6 (**C**), *n* = 3–5 (**D**–**F**). ** *p* < 0.01, *** *p* < 0.001, **** *p* < 0.0001.

The results of the BrdU assay showed that GPER1 agonist G1 treatment decreased proliferation of the vulvar carcinoma cells A431 and CAL-39. A slight inhibitory effect was observed in A431 cells treated with 1 μM G1 (M = 90.026 ± 6.95; *n* = 4; *p* = 0.3328) but was dose-dependently significant with 2.5 μM (M = 71.475 ± 2.387; *n* = 4; *p* = 0.0003) and 5 μM (M = 47.267 ± 3.785; *n* = 4; *p* < 0.0001) (Figure 4A). In CAL-39 cells, the inhibition of proliferation was already significant at treatment with 1 μM G1 (M = 70.021 ± 7.221; *n* = 4; *p* < 0.0001) and dose-dependently stronger at 2.5 μM (M = 24.226 ± 3.601; *n* = 4; *p* < 0.0001) and 5 μM (M = 16.885 ± 3.056; *n* = 4; *p* < 0.0001) (Figure 4B). The GPER1 antagonist G36 did not have any significant effect on the vulvar carcinoma cells at all doses compared to the ethanol control (Figure 4A,B). Inhibition of GPER1 with G36 at concentrations of 0.5–5 μM had no significant effect on the viability of the carcinoma cells in either cell line (Figure 4C,D). The GPER1 agonist G1 led to a dose-dependent decrease in cell viability in both cell lines. In A431, a slight reduction in cell viability was achieved at doses of 0.5 μM G1 (M = 93.72 ± 1.745; *n* = 5; *p* = 0.3809) but a significant reduction at doses of 1.25 μM (M = 64.4 ± 10.037; *n* = 3; *p* < 0.0001). This effect was dose-dependently amplified in G1 for concentrations 2.5 μM (M = 13.05 ± 1.429; *n* = 4; *p* < 0.0001) and 3.75 μM (M = 3.167 ± 0.338; *n* = 3; *p* < 0.0001) (Figure 4E). In CAL-39 cells, a significant reduction in cell viability was already achieved at 0.5 μM G1 (M = 72.25 ± 9.962; *n* = 4; *p* = 0.0069) but stronger at 1.25 μM (M = 28.333 ± 8.685; *n* = 3; *p* < 0.0001) and 2.5 μM (M = 15.3 ± 5.081; *n* = 4; *p* < 0.0001) with the minimum measured viability being almost reached at 3.75 μM (M = 13.167 ± 0.731; *n* = 3; *p* < 0.0001) (Figure 4F). Compared to the control in both cell lines, no significant effect of ethanol treatment on cell viability was observed (Figure 4C–F).

### 2.6. Signs of Necrosis after Treatment with GPER1 Agonist G1

**Figure 5:**
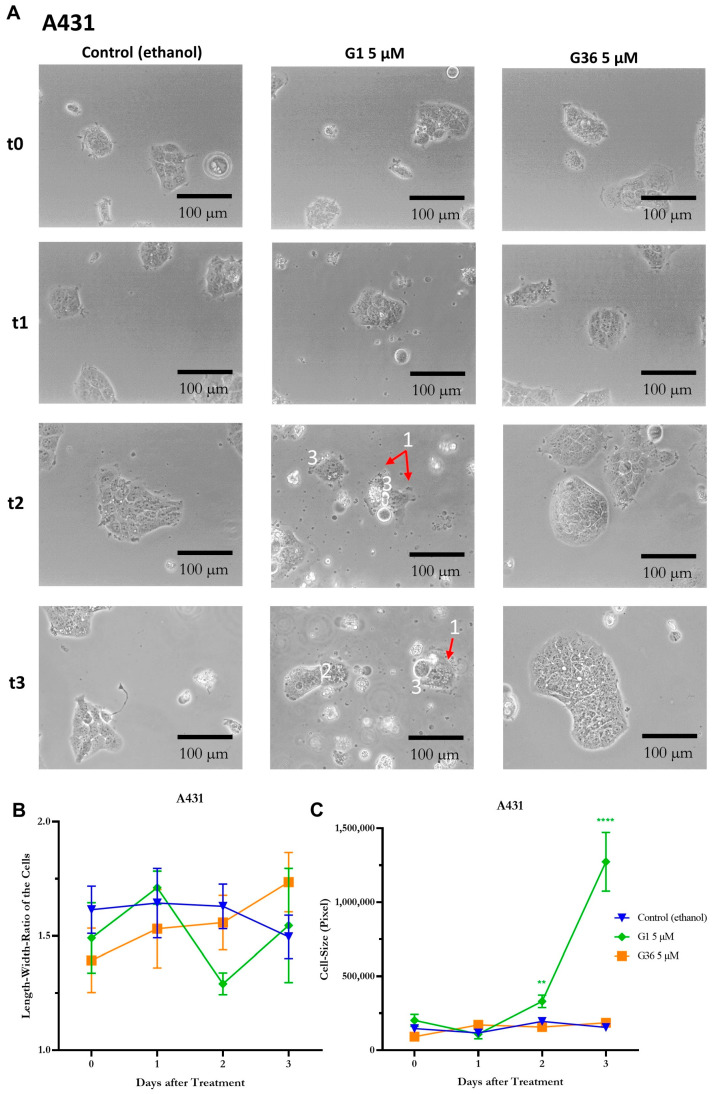
Signs of necrosis after treatment with GPER1 agonist G1 in vulvar carcinoma A431. Photos of the cancer cells after treatment with G1 or G36 vs. control (ethanol) at time t0 (treatment)–t3 (72 h after treatment), interval 24 h (**A**). Necrosis signs: 1 = cell contents are released; 2 = karyolysis; 3 = cell detaches from cell association, cell swelling (**C**). Changes after treatment in length-to-width ratio (**B**) and cell size (**C**). Mean with SEM, one-way ANOVA, 6–12 cells per treatment, *n* = 3 (**B**,**C**). Magnification 20×. ** *p* < 0.01, **** *p* < 0.0001.

In the A431 vulvar carcinoma cells, signs of necrosis such as release of cell contents, karyolysis, cell separation from the cell association, and cell swelling were observed after treatment with G1 at time-points t2 (48 h after treatment) and t3 (72 h after treatment). No similar effects were observed after treatment with ethanol (control) and G36 (Figure 5A). The statistical analysis of the cell’s length–width ratio did not show any significant differences at all time points and treatments. The ratio was about 1.5 (Figure 5B). The size of the cells remained nonsignificant compared to the ethanol control after G36 treatment, but it increased significantly at t2 (M = 330,370.667 ± 42,431.936; *n* = 6; *p* = 0.0032) and t3 (M = 1,273,521 ± 198,552.721; *n* = 6; *p* < 0.0001) after G1 treatment (Figure 5C).

**Figure 6:**
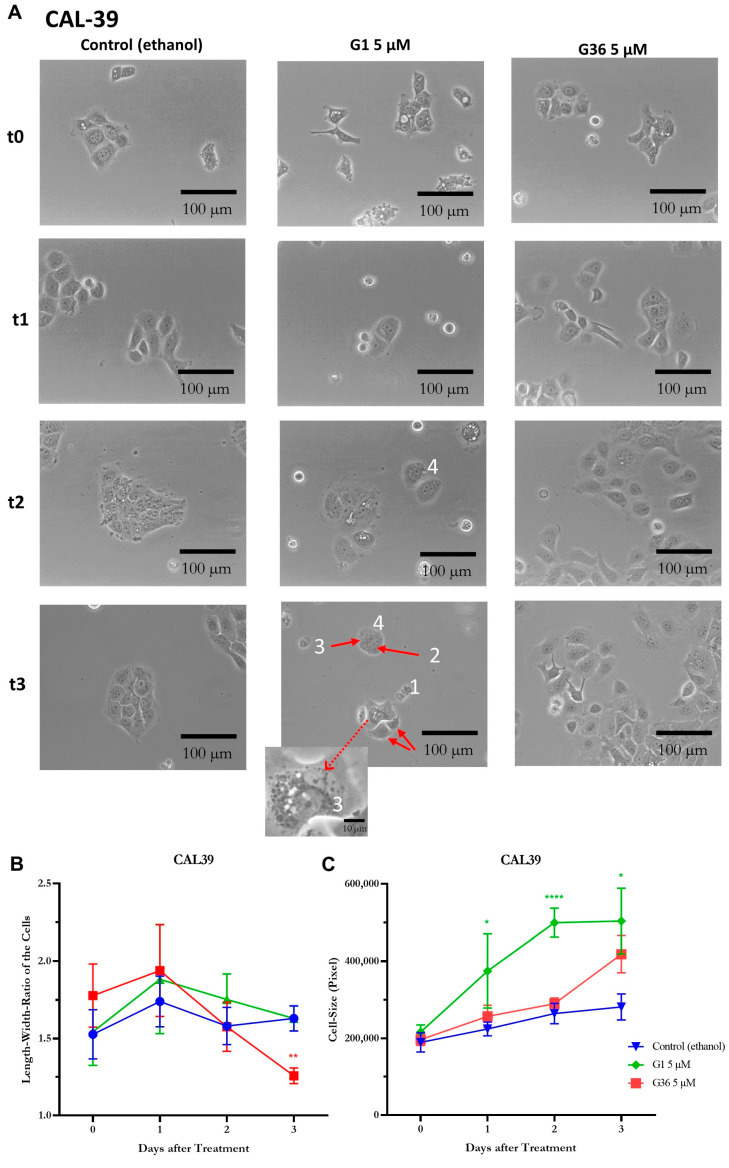
Signs of necrosis after treatment with GPER1 agonist G1 in vulvar carcinoma cells CAL-39. Photos of the cancer cells after treatment with G1/G36 vs. control (ethanol) at time t0 (treatment)–t3 (72 h after treatment), interval 24 h (**A**). Necrosis signs: 1 = cell contents are released; 2 = karyolysis; 3 = chromatin condenses and clumps to nuclear membrane; 4 = cell detaches from cell association, cell swelling (**C**). Changes after treatment in length-to-width ratio (**B**) and cell size (**C**). Mean with SEM, one-way ANOVA, 6–12 cells per treatment, *n* = 3 (**B**,**C**). Magnification 20×. * *p* < 0.05, ** *p* < 0.01, **** *p* < 0.0001.

In the CAL-39 cell line, the signs of necrosis—release of cell contents, karyolysis, chromatin condensation and clumping to nuclear membrane, cell separation from the cell association, and cell swelling—were observed after G1 treatment, starting slightly at t2 and unambiguous at t3 (Figure 6A). No similar effects were observed after ethanol (control) and G36 treatment (Figure 6C). The length–width ratio of the cells remained not significantly different after ethanol (control) and G36 treatment at all time points. After G1 treatment, a significantly lower length–width ratio was achieved at t3 (M = 1.63 ± 0.081; *n* = 12; *p* = 0.0069) compared to the ethanol control (Figure 6B). The cell size was significantly increased compared to the ethanol control after G1 treatment at time-points t1 (M = 374,616.125 ± 95,908.505; *n* = 8; *p* = 0.0482), t2 (M = 499,313.7 ± 37,611.973, *n* = 10; *p* < 0.0001), and t3 (M = 503,506.111 ± 85,248.282; *n* = 9; *p* = 0.0149) (Figure 6C).

### 2.7. Effects of GPER1 Agonist G1 and Antagonist G36 on Migration of Vulvar Carcinoma Cells

**Figure 7:**
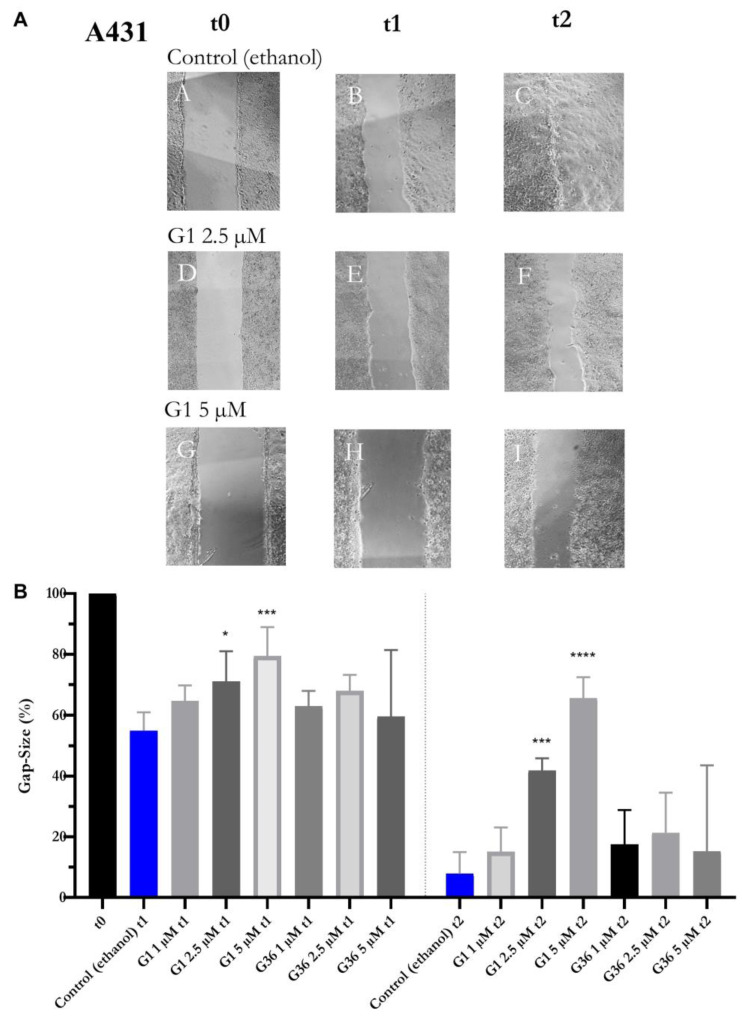
Inhibition of migration of vulvar carcinoma cells A431 by GPER1 agonist G1. Photos of the gaps after treatment with G1 vs. control (ethanol) at time t0–t2, interval 10 h (**A**). Presentation of the effects of treatment with G1 or G36 vs. control (ethanol) on migration (**B**). Mean with SEM, one-way ANOVA, compared to control (ethanol), *n* = 5–7 (B). Magnification 4×. * *p* < 0.05, *** *p* < 0.001, **** *p* < 0.0001.

**Figure 8:**
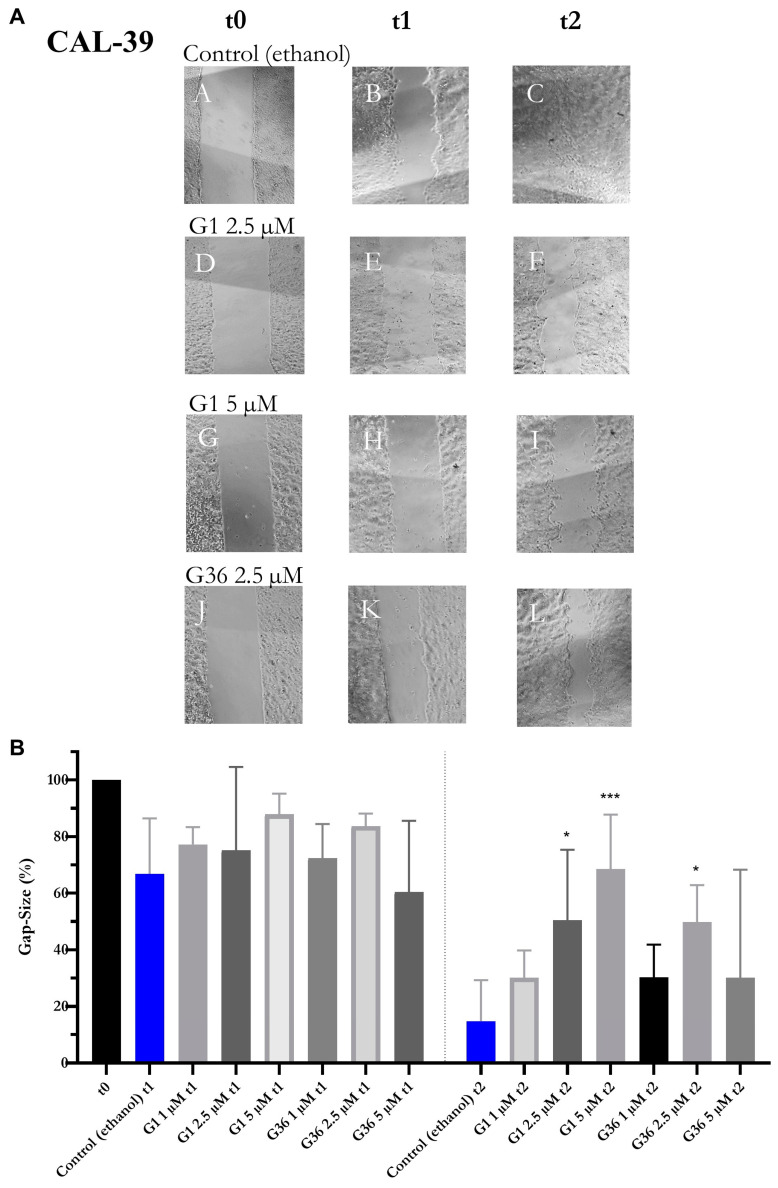
Inhibition of migration of vulvar carcinoma cells CAL-39 by GPER1 agonist G1 and antagonist G36. Photos of the gaps after treatment with G1 or G36 vs. control (ethanol) at time t0–t2, interval 10 h (**A**). Effects of treatments with G1 or G36 vs. control (ethanol) on migration (**B**). Mean with SEM, one-way ANOVA, *n* = 4–7 (**B**). Magnification 4×. * *p* < 0.05, *** *p* < 0.001.

G36 treatment at all concentrations did not result in any significant changes in gap size or migration compared to the ethanol control at both time-points t1 and t2 on A431 cells. Treatment with 1 μM G1 did not affect the migration of A431 cells. G1 inhibited the migration of A431 cells at t1 at 2.5 μM (M = 71.008 ± 4.082, *n* = 6; *p* = 0.0210) and 5 μM (M = 79.438 ± 4.251, *n* = 5; *p* = 0.0005). This inhibition was further amplified at t2 after treatment with G1 at concentrations of 2.5 μM (M = 41.833 ± 1.644, *n* = 6; *p* = 0.0001) and 5 μM (M = 65.593 ± 3.078, *n* = 5; *p* < 0.0001) (Figure 7B), as demonstrated in the photographs (Figure 7A). The migration of the CAL-39 vulvar carcinoma cells was not significantly affected by G1 and G36 treatment at t1 compared to the ethanol control. At t2, treatment with G1 slowed gap closure at 2.5 μM (M = 50.488 ± 11.119, *n* = 5; *p* = 0.0255) and 5 μM (M = 68.572 ± 7.828, *n* = 6; *p* = 0.0002). There was no significant effect at lower concentrations. G36 treatment at 1 μM and 5 μM had no observable effects while at 2.5 μM (M = 49.803 ± 4.933, *n* = 7; *p* = 0.0145) it reduced migration compared to the ethanol control (Figure 8B). The photographs of the treatments with significant effects on migration are shown in Figure 8A.

### 2.8. Effects of GPER1 Agonist G1 and Antagonist G36 on Colony Formation and Sphere Formation

**Figure 9:**
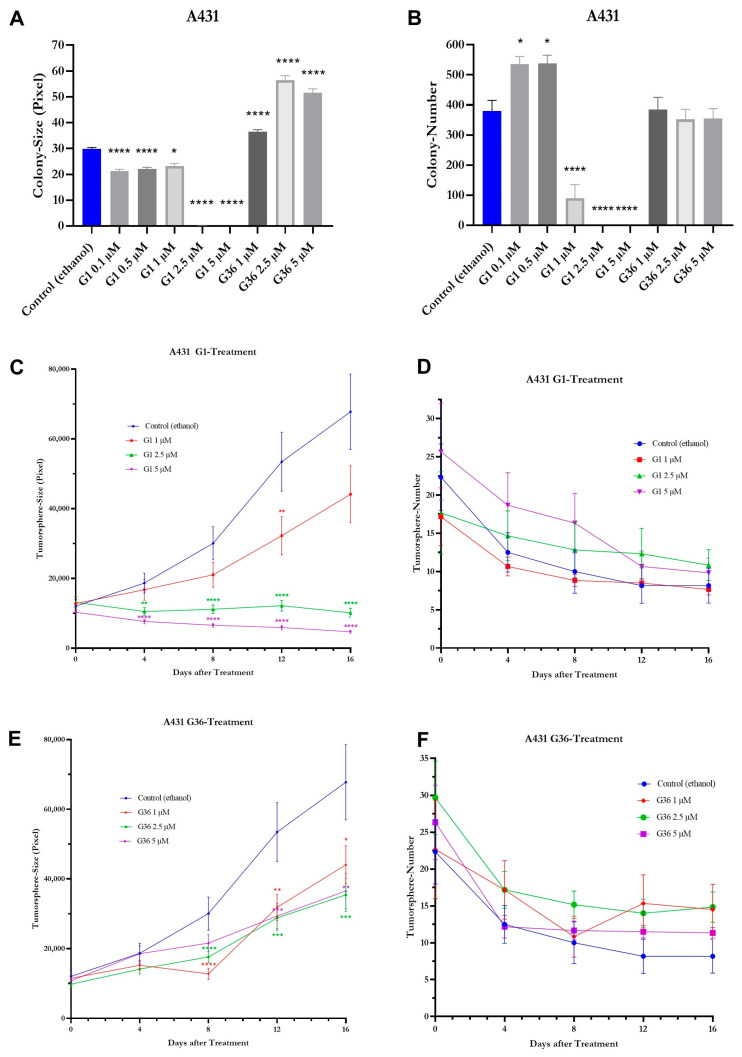
Effects of GPER1 agonist G1 and antagonist G36 on colony formation and tumor sphere formation in vulvar carcinoma cells A431. Presentation of the effects of treatment with G1/G36 vs. control (ethanol) on colony size (**A**) and number (**B**), and on tumor sphere size (**C**,**E**) and number (**D**,**F**). Mean with SEM (**A**–**F**), one-way ANOVA (**A**–**F**), *n* = 81–2312 (**A**), *n* = 3–9 (**B**), *n* = 45–160 (**C**,**E**), *n* = 5 (**D**,**F**). * *p* < 0.05, ** *p* < 0.01, *** *p* < 0.001, **** *p* < 0.0001.

In the vulvar carcinoma cell line A431, the colony size was significantly reduced compared to the ethanol control following treatments with G1 at concentrations of 0.1 μM (M = 21.21 ± 1.503, *n* = 1611; *p* < 0.0001), 0.5 μM (M = 22.1 ± 1.501, *n* = 1617; *p* < 0.0001), 1 μM (M = 23.23 ± 2.294, *n* = 545; *p* = 0.0336), 2.5 μM (M = 0.09968 ± 5.229, *n* = 93; *p* < 0.0001), and 5 μM (M = 0 ± 5.593, *n* = 81; *p* < 0.0001). The colony size was significantly increased compared to the ethanol control following treatment with G36 at concentrations of 1 μM (M = 36.46 ± 1.339, *n* = 2312; *p* < 0.0001) and 5 μM (M = 51.58 ± 1.365, *n* = 2168; *p* < 0.0001), with the strongest effect at 2.5 μM (M = 56.49 ± 1.375, *n* = 2119; *p* < 0.0001) (Figure 9A). After treatment with G1, the colony number was significantly increased compared to the ethanol control at concentrations of 0.1 μM (M = 536 ± 25.239, *n* = 3; *p* = 0.0394) and 0.5 μM (M = 538 ± 27.429, *n* = 3; *p* = 0.0359), but was dose-dependent significantly reduced at concentrations of 1 μM (M = 90.333 ± 44.867, *n* = 6; *p* < 0.0001), 2.5 μM (M = 0.167 ± 0.167, *n* = 6; *p* < 0.0001), and 5 μM (M = 0 ± 0, *n* = 6; *p* < 0.0001). The colony number was not significantly different from the ethanol control following treatment with G36 at all concentrations (Figure 9B).

The size of tumor spheres was compared to the ethanol control after treatment of G1 at 1 μM on day 8 (M = 21,048.245 ± 3584.927, *n* = 53; *p* = 0.0567) and day 16 (M = 44,125.283 ± 8145.794, *n* = 46; *p* = 0.0508) tendentiously reduced but significantly reduced on day 12 (M = 32,251.216 ± 5503.460, *n* = 51; *p* = 0.0073). At 2.5 μM G1, the size of tumor spheres was significantly reduced on day 4 (M = 10514.057 ± 1105.903, *n* = 88; *p* = 0.0086), day 8 (M = 11,154.221 ± 1203.315, *n* = 77; *p* < 0.0001), day 12 (M = 10,156.815 ± 1330.249, *n* = 74; *p* < 0.0001), and day 16 (M = 10,156.815 ± 1330.249, *n* = 65; *p* < 0.0001). The size of tumor spheres was also significantly reduced at 5 μM G1 on day 4 (M = 7662.938 ± 678.615, *n* = 112; *p* < 0.0001), day 8 (M = 6587.735 ± 544.729, *n* = 98; *p* < 0.0001), day 12 (M = 5922.547 ± 642.367, *n* = 64; *p* < 0.0001), and day 16 (M = 4698.797 ± 374.268, *n* = 59; *p* < 0.0001) (Figure 9C).

During the observation period, the number of tumor spheres did not differ between ethanol (control), G1, and G36 treatment (Figure 9D,F). The size of tumor spheres was significantly reduced after treatment with G36 at a concentration of 1 μM on day 8 (M = 12,784.092 ± 1625.408, *n* = 65, *p* < 0.0001), day 12 (M = 31,820.967 ± 3700.883, *n* = 92; *p* = 0.0014), and day 16 (M = 44,043.552 ± 5480.897, *n* = 87; *p* = 0.0183), after treatment with 2.5 μM on day 8 (M = 17,626.044 ± 1580.861, *n* = 91; *p* < 0.0001), day 12 (M = 28,805.060 ± 3518.959, *n* = 84; *p* = 0.0003), and day 16 (M = 35,438.506 ± 4760.253, *n* = 89; *p* = 0.0005), and after treatment with 5 μM on day 12 (M = 29,327.087 ± 3657.152, *n* = 69; *p* = 0.0006) and day 16 (M = 36,558.309 ± 5034.015, *n* = 68; *p* = 0.0016) (Figure 9E).

**Figure 10:**
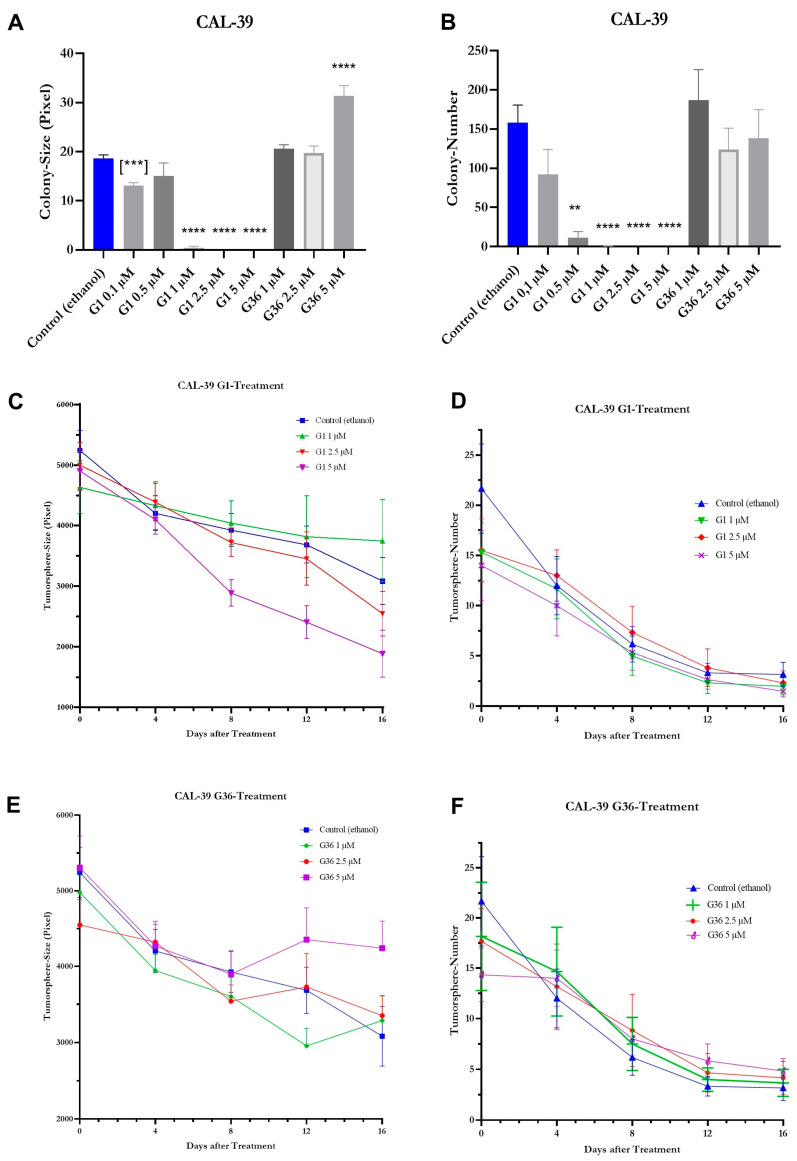
Effects of GPER1 agonist G1 and antagonist G36 on colony formation and tumor sphere formation in vulvar carcinoma cells CAL-39. Plots of the effects of treatment with G1/G36 vs. control (ethanol) on the colony size (**A**) and number (**B**), and on tumor sphere size (**C**,**E**) and number (**D**,**F**). Mean with SEM (**A**–**F**), one-way ANOVA (**A**–**F**), (***) = unpaired *t*-test (**A**), *n* = 35–1121 (**A**), *n* = 3–9 (**B**), *n* = 11–164 (**C**,**E**), *n* = 5 (**D**,**F**). ** *p* < 0.01, *** *p* < 0.001, **** *p* < 0.0001.

The colony size of CAL-39 vulvar carcinoma cells was significantly reduced after treatment with 0.1 μM G1 (M = 13.07 ± 2.404, *n* = 273; *p* = 0.0005) compared to the ethanol control. After treatment with 0.5 μM G1 (M = 15.06 ± 6.224, *n* = 35; *p* = 0.9973), a nonsignificantly reduced colony size was observed. The reduction in colony size was significantly increased compared to the ethanol control after treatment with G1 at concentrations of 1 μM (M = 0.4167 ± 3.836, *n* = 96; *p* < 0.0001), 2.5 μM (M = 0 ± 3.855, *n* = 95; *p* < 0.0001), and 5 μM (M = 0 ± 3.855, *n* = 95; *p* < 0.0001). At concentrations of 1 μM and 2.5 μM, GPER1 antagonist G36 had no effect on colony size. At 5 μM, G36 (M = 31.34 ± 1.95, *n* = 829; *p* < 0.0001) increased the colony size significantly compared to the ethanol control (Figure 10A). A slight decrease in colony number compared to the ethanol control was observed after treatment with G1 at a concentration of 0.1 μM (M = 92 ± 31.974, *n* = 3; *p* = 0.5022) and significantly reduced colony number after treatment with 0.5 μM (M = 11.667 ± 7.688, *n* = 3; *p* = 0.0053), 1 μM (M = 0.333 ± 0.333, *n* = 6; *p* < 0.0001), 2.5 μM (M = 0 ± 0, *n* = 6; *p* < 0.0001), and 5 μM (M = 0 ± 0, *n* = 6; *p* < 0.0001). Treatment with G36 at all the concentrations used did not significantly affect the number of colonies (Figure 10B). Regardless of treatment with G36, G1, or ethanol (control), the tumor spheres of CAL-39 cells that formed were consistently dissolved on the ultralow attachment plate. Accordingly, without a significant difference compared to the ethanol control, the number of tumor spheres steadily decreased after treatment with G1 or G36 at the concentrations noted (Figure 10D,F). During the period examined, there were no significant differences in the size of the tumor spheres after treatment with G1 and G36 when compared to ethanol control (Figure 10C,E).

## 3. Discussion

GPER1 is often involved in the progression of neoplasms with varying impacts. Its role as a tumor suppressor or an oncogene appears to depend on the tissue type and tumor entity [15]. The precise effects of GPER1 in carcinogenesis remain unclear and are subject to discussion within specific tissues [10,27]. This study aims to examine the function of GPER1 in vulvar carcinoma.

Using immune cytochemistry, which allows the visualization of molecular markers [31], it has been demonstrated that GPER1 is expressed in the cytoplasm and nuclei of vulvar carcinoma cells A431 and CAL-39. The cytoplasmic localization of GPER1 in the A431 cell line has also been validated by Lan et al. [12]. GPER1 has been localized in the cytoplasm and nucleus of cervical neoplasms [10] and at the cell membrane, particularly in the area of invasion fronts [11].

Tissue microarrays enable the quantification and localization of proteins [32]. The staining of the microarray indicates that GPER1 is consistently expressed in vulvar carcinoma, as evidenced by the presence of GPER1 staining in all samples. The receptor is primarily observed in the epithelial layers or in the stroma with infiltrating tumor cells of the tissue samples. Additionally, a high immune-reactive value, which corresponds to elevated GPER1 expression, showed a positive correlation with a high tumor grade. Hence, similar findings have been observed in cervical carcinoma [26,29]. Accordingly, this high GPER1 expression is prognostically associated with lower 5-year survival rates in patients with cervical carcinoma [11]. Likewise, in breast carcinoma, a correlation has been observed between high GPER1 expression, increased cell proliferation, invasion, migration [23], and worsened prognosis [21].

To analyze the impact of GPER1 activation or inhibition on vulvar carcinoma cells, various aspects such as cell viability, proliferation, migration, colony formation, and sphere formation were examined following treatment with the GPER1 agonist G1 or the GPER1 antagonist G36. Although G1 is considered to be a selective GPER1 agonist [9], receptor-independent effects have been demonstrated by showing that G1 is able to interact directly with tubulin [33,34,35] at the same site as colchicine [33]. Therefore, we checked whether the biological effect of G1 occurs through GPER1 in vulvar carcinoma cells. We could demonstrate that the effect of G1 was abolished in a dose-dependent manner in the presence of GPER1 antagonist G36 (not shown). This implies that G1 mediates its effects via binding to GPER1. This does not exclude additional receptor-independent effects.

The BrdU assay was used to assess the proliferation of the cells [36]. Results revealed a dose-dependent decrease in proliferation at 1 μM G1 for CAL-39 and 2.5 μM for A431. Han et al. [20] reported similar findings for the G1 impact in ovarian carcinoma cells. GPER1 activation also leads to reduced proliferation in breast carcinoma cells [20], while in contrast, Girgert et al. [37] observed decreased growth in triple-negative breast carcinoma cells following GPER1 knockdown. Correspondingly, inhibiting GPER1 resulted in decreased proliferation in experiments involving oral squamous cell carcinoma [13]. 

During the evaluation of cell viability using the Resazurin assay [38], activating GPER1 with at least 0.5 μM G1 in CAL-39 and 1.25 μM in A431 resulted in a decline in cell viability. Han et al. [20] also observed reduced cell viability in ovarian carcinoma cells CaOV3 and CaOV4 after treatment with G1, as assessed by an MTT assay. In contrast, Lan et al. [12] found that treatment of A431 cells with 0.01–0.5 μM G1 led to a dose-dependent increase in viability. Furthermore, a decrease in cell viability was observed after treatment with 5 μM GPER1 antagonist G15 [12]. Since the same A431 cell line was used by Lan et al. [12] as in this study, cell-line-specific factors can be ruled out as the cause of the contradictory results. It is possible this different effect can be explained with the use of GPER1 antagonist G15 instead of G36, as G15 has the ability to bind to ERα and ERβ, which is not the case for G36 [17]. This could have activated other signaling pathways than treatment with G36.

The migration ability of vulvar carcinoma cells was investigated using the gap closure assay [39]. For A431 cells, it was dose-dependently reduced by GPER1 activation with at least 2.5 μM G1 after 10 h, while CAL-39 cells exhibited a reduction after 20 h. Likewise, the migration ability of ovarian carcinoma cells was reduced by G1 in a wound-healing scratch-assay [20].

The colony formation ability is influenced by cell proliferation and migration. The impact of GPER1 activation/inhibition on this ability of vulvar carcinoma cells was assessed using the colony formation assay [40]. In A431 cells, the activation of GPER1 with 0.1 μM and 0.5 μM G1 led to an increase in colony number, followed by a dose-dependent decrease from 1 to 5 μM G1. Lower doses may have caused colony disintegration, resulting in reduced colony size but increased count. At 1 μM G1, the tumor-suppressive effect became more prominent, oppressing the formation of new colonies. This is supported by the observation that even at 0.1 μM G1 colony size was reduced compared to the control, but a significant decrease first was achieved at 2.5 μM G1. In CAL-39 cells, treatment with G1 resulted in a dose-dependent decrease in colony number and size. These results support a tumor-suppressive function of GPER1 activation. 

To observe cell proliferation and their ability to form tumor spheres, the sphere formation assay was used [41]. Treatment with G1 and G36 did not affect the number of tumor spheres. In A431 cells, however, the size of tumor spheres decreased in a dose-dependent manner after treatment with G1. Thus, it can be inferred that GPER1 activation has a tumor-suppressive effect in this assay. In CAL-39 cell line, both the number and size of tumor spheres decreased without significant differences during the observation period in the G1-treated, G36-treated, and control. It is likely that CAL-39 cells faced challenges in forming and maintaining stable tumor spheres in the nonadherent environment, which explains the lack of treatment differences.

To gain a better understanding of the effects of G1 and G36 on the cells, cell morphology was observed after treatment. In A431 cells, signs of necrosis such as cell content release, significant cell swelling, cell detachment from the cell cluster, and karyolysis were observed after G1 treatment. Additionally, chromatin condensation at the nuclear membrane was seen in CAL-39 cells. Consequently, GPER1 activation leads to necrosis, a nonprogrammed cell death. These observations support the tumor-suppressive effect of GPER1 shown in other experiments. Hernandez-Silva et al. [10] also reported an increase in necrosis and apoptosis after G1 treatment in cervical neoplasia.

By conducting Western blot analysis, changes in protein expression of estrogen receptors in vulvar carcinoma cells A431 and CAL-39 were examined after GPER1 activation/inhibition. However, no significant alterations in expression were observed, suggesting that the effects of G1 and G36 treatment shown in this study were not caused by changes in the expressions of GPER1, ERα, or ERβ. 

In the gap closure assay, migration of CAL-39 vulvar carcinoma cells was inhibited after treatment with 2.5 μM G36, similar to the inhibitory effect observed after G1 treatment. An increased or decreased expression of estrogen receptors cannot be a reason for that. In the colony formation assay, it was noticed that G36 treatment increased the size of colonies in A431 cells at all doses and in CAL-39 cells at only 5 μM, while the number of colonies remained unchanged compared to the control. This suggests that growth occurred within existing colonies. However, it is contradictory that no changes in cell size or morphology were observed compared to the control in the examination of cell morphology and the BrdU assay showed no effects from G36 treatment on proliferation. Surprisingly, the size of A431 tumor spheres decreased compared to the control after G36 treatment. This decrease was independent of the dosage. Further, the size difference to the control remained relatively constant instead of increasing with higher doses as expected with inhibition. The splitting of the spheres cannot explain these observations since the number of tumor spheres did not increase either. The inhibition of GPER1 using G36 did not yield significant results in the other experiments of this study.

## 4. Materials and Methods

### 4.1. Cell Culture

The human vulvar cancer cell lines A431 and CAL-39 were obtained from American Type Cell Collection (ATCC; Manassas, VA, USA). They were cultured in Minimum Essential Medium (MEM; Thermo Fisher Scientific, Waltham, MA, USA) supplemented with 0.1% Transferrin (Sigma-Aldrich, St. Louis, MO, USA), 1% Penicillin/Streptomycin (*p*/S; Thermo Fisher Scientific), 10% fetal calf serum (FCS; Pan Biotech, Aidenbach, Germany), and 26 IU Insulin (Sanofi, Paris, France), in a humidified atmosphere with 5% CO_2_ at 37 °C.

### 4.2. Drugs

GPER1 agonist G1 and GPER1 antagonist G36 were purchased from Biomol (Hamburg, Germany). G1 and G36 were dissolved in ethanol. Each control was treated with 0.03 *v*/*v*% ethanol.

### 4.3. Proliferation

The BrdU assay was implemented with a kit (Roche Applied Science, Penzberg, Germany). Five thousand cells per well were seeded in 96-well plates (Corning Life Sciences, Tewksbury, MA, USA). They were treated with G1 or G36 after 24 h and incubated for 50 h. The cells were treated with 10 µL BrdU Labeling Reagent 1:100 in MEM. After another 22 h of incubation, the BrdU amount was measured by a multidetection microplate reader (BioTek Instruments, Winooski, VT, USA) and GEN5 3.12 software (BioTek Instruments).

### 4.4. Viability

For the Resazurin assay, 600 cells of A431 and 1200 cells of CAL-39 per well were seeded in 96-well plates (Corning Life Sciences) in Dulbecco’s Minimum Essential Medium w/o phenol-red (DMEM; Thermo Fisher Scientific). A greater quantity of CAL-39 cells was utilized to ensure that the color change occurred simultaneously in both cell lines, enabling a simultaneous execution of the assay. The treatment with G1 or G36 was carried out 24 h later. After incubation for 72 h, 20 µL Resazurin (Thermo Fisher Scientific) was added to every well. Ten hours later, the relative reduction in Resazurin was measured at 570 nm and 630 nm using a multidetection microplate reader (BioTek Instruments) and analyzed using GEN5 1.08 software (BioTek Instruments). The relative reduction was calculated with Excel (Microsoft, Redmond, WA, USA).

### 4.5. Cell Morphology 

In a 6-well plate (Greiner Bio-One, Frickenhausen, Germany), 30,000 cells per well of A431 and 40,000 cells per well of CAL-39 were seeded. The cells were treated 24 h later with 5 µM G36 or G1. The software uEye Cockpit 2.0 (IDS Imaging Development Systems, Obersulm, Germany) was used to obtain pictures of the wells at t0–t3, with t0 after treatment and the others in a 24 h interval. The cells were analyzed with ImageJ (Wayne Rasband, National Institutes of Health, Bethesda, MD, USA).

### 4.6. Gap Closure

One hundred forty thousand cells per well of A431 and CAL-39 were seeded in a 24-well plate (Greiner Bio-One) separated by an insert. After incubation for 24 h, the cells were washed with Dulbecco’s Phosphate Buffered Saline (DPBS; Pan Biotech) and treated with G1/G36. Pictures of the cells were taken using uEye Cockpit 2.0 (IDS Imaging Development Systems, Obersulm, Germany) at t0 and every 10 h until the gap was closed. The gap closure was analyzed by using Adobe Photoshop CS5 12.0 (Adobe Inc., San José, CA, USA), ImageJ 1.52a (Wayne Rasband), and Microsoft Excel (Microsoft). 

### 4.7. Colony Formation

One thousand cells of A431 and CAL-39 per well were seeded in a 6-well plate (Greiner Bio-One). After 24 h, the cells were treated with G1 or G36 and incubated for five to seven days until the colonies emerged. The plates were stained with crystal violet (Sigma-Aldrich) and scanned using Epson Perfection V850 pro (Epson, Suwa, Japan) and the Epson Scan 2 software (Epson). Colonies were analyzed with ImageJ 1.52a (Wayne Rasband).

### 4.8. Tumor Sphere Formation 

One thousand cells per well were seeded in an ultralow attachment 96-well plate with flat bottom (Corning Life Sciences). After 24 h of incubation, the cells were treated with G1/G36. Using the Celigo Cyntellect cell imaging cytometer (Nexcelom Bioscience LLC, Lawrence, KS, USA) and the Celigo 2.1.0.96 software (Celigo Inc., San Mateo, CA, USA), the plates were photographed every 4 days, starting after treatment with t0 until t5. The tumor spheres were analyzed using ImageJ 1.52a (Wayne Rasband).

### 4.9. Tissue Microarray

The vulvar carcinoma tissue microarray (US Biomax, Derwood, MD, USA) was deparaffinized, unmasked, and incubated with rabbit antihuman GPER1 antibody at 1:200 dilution (Thermo Fisher Scientific) overnight in a wet chamber. The next day, after washing, it was incubated with Alexa Fluor 488 antirabbit IgG antibody solution (Invitrogen, Waltham, MA, USA) at 1:10 dilution. Nuclei were stained using 4′,6-diamidino-2-phenylindole dihydrochloride at 1:1000 dilution (DAPI; Novus Biologicals, Littleton, CO, USA). The tissue microarray was embedded with fluorescence mounting medium (Dako North America, Carpinteria, CA, USA). Pictures were taken using the software cellSens Dimension 3.2 (Olympus Life Science Solutions, Tokyo, Japan) and an Olympus IX83microscope (Olympus Life Science Solutions).

### 4.10. Immune Cytology 

Using an 8-well plate on glass (Sarstedt, Nümbrecht, Germany), 4000 and 8000 cells of A431 and 12,000 and 16,000 cells of CAL-39 were seeded and incubated for 24 h. After incubation with formaldehyde (3.7%; Sigma-Aldrich) for 15–30 min, the cells were washed twice with fluorescence staining solution/DPBS (Pan Biotech), 2% BSA (Carl Roth, Karlsruhe, Germany) and 0.25% Triton X-100 (Sigma-Aldrich). The cells were incubated overnight with the rabbit antihuman GPER1 antibody at 1:100 dilution (Thermo Fisher Scientific) in a wet chamber. After washing, fluorescence staining solution containing DAPI at 1:1000 dilution (Novus Biologicals) and Alexa Fluor 488 antirabbit IgG antibody solution (Invitrogen) at 1:10 dilution were added. After 30 min incubation, the cells were washed twice with fluorescence staining solution and once with DPBS. Fluorescence mounting medium (Dako North America) was added and covered with a cover slip (Menzel-Gläser, Braunschweig, Germany) and allowed to dry. The stained cells were photographed using an Olympus IX83 microscope (Olympus Life Science Solutions) and cellSens Dimension 3.2 software (Olympus Life Science Solutions). 

### 4.11. Western Blot

Initially, 250,000 cells of A431 and 500,000 cells of CAL-39 were seeded and incubated for 24 h. The cells were then treated with G36 and G1 and incubated for 72 h. After washing the cells with DPBS (Pan Biotech), they were incubated 15–20 min with PBS-EDTA (100 mL DPBS, 200 mg EDTA tetra sodium salt (Affymetrix, Cleveland, OH, USA), detached, and centrifuged at 1300× *g* rcf for 5 min. Then, they were lysed in lytic buffer (Sigma-Aldrich, St. Louis, USA), 100 µL phosphatase inhibitor (Sigma-Aldrich, St. Louis, MO, USA), and 100 µL protease inhibitor (Sigma-Aldrich, St. Louis, MO, USA). Protein concentration was measured by the Bradford method using a multidetection microplate reader (BioTek Instruments) at 590 nm and GEN5 1.08 software (BioTek Instruments). Protein was diluted 1:1 with Laemmli buffer (Fresenius Kabi, Bad Homburg vor der Höhe, Germany), 5 mL LDS sample buffer (Thermo Fisher Scientific), and 1 mL NuPage sample-seducing agent (Thermo Fisher Scientific). The proteins were fractioned using SDS-PAGE and Western blot apparatus (Bio-Rad Laboratories, Hercules, CA, USA). The membrane was incubated overnight at 4 °C with rabbit antihuman GAPDH at 1:1000 dilution (Cell Signaling Technology, Danvers, MA, USA), rabbit antihuman GPER1 at 1:2000 dilution (Thermo Fisher Scientific), rabbit antihuman ERα at 1:200 dilution from a 100 μg/mL stock solution (Santa Cruz Technology, Dallas, TX, USA), or rabbit antihuman ERβ at 1:200 dilution from a 100 μg/mL stock solution (Santa Cruz Technology). Before incubating with antirabbit IgG at 1:10,000 or 1:40,000 (GAPDH) dilution (Dianova, Hamburg, Germany) for 1 h, the membrane was washed three times for 10 min with TBST (0.1% Tween-20 (AppliChem, Darmstadt, Germany) in TBS). Chemiluminescence solution (400 µL peroxide solution (Westar Supernova, Cyanagen, Italy) and 400 µL Luminol amplification solution (Westar Supernova)) was applied to the membrane for 1 min. The Western blots were detected using a C-Digit blot scanner (Li-Cor Biosciences, Lincoln, NE, USA) and analyzed with Image Studio 4.0 software (Li-Cor Biosciences).

### 4.12. Statistical Analysis

The experiments were performed on at least three technical and biological replicates. Data was statistically analyzed using one-way ANOVA or rarely unpaired, two-tailed, parametric *t*-tests by GraphPad Prism 8.0.1 (GraphPad Software, San Diego, CA, USA). In the figures presented, the significances are represented by * (*p* < 0.05), ** (*p* < 0.01), *** (*p* < 0.001), and **** (*p* < 0.0001).

## 5. Conclusions

Based on the findings of this study, the activation of GPER1 in vulvar carcinoma appears to have a tumor-suppressive effect. This is supported by the decrease in migration, proliferation, colony formation, and tumor sphere formation, together with the presence of morphological signs of necrosis and the reduction in cell viability after G1 treatment in both vulvar carcinoma cell lines. In the CAL-39 cell line compared to the A431 cell line, these effects were observed at lower doses in the Resazurin, BrdU, colony formation, and sphere formation assays. Changes in expression of estrogen receptors do not seem to be the underlying cause of these observations. Additionally, there may be a correlation between GPER1 expression and malignancy grade of vulvar carcinoma. Further evidence is needed to better understand the function of GPER1 in vulvar carcinoma and to potentially utilize GPER1 activation in medical treatment.

## Data Availability

The datasets used and/or analyzed during the current study are available from the corresponding author on reasonable request.

## Supplementary Materials

The following supporting information can be downloaded at: https://www.mdpi.com/article/10.3390/ijms241813705/s1.
