# Supplementary material for: Activation of G-Protein-Coupled Estrogen Receptor 1 (GPER1) Reduces Progression of Vulvar Carcinoma Cells
Source: Int J Mol Sci. 2023 Sep 5;24(18):13705. doi: 10.3390/ijms241813705 (PMC10530864; doi:10.3390/ijms241813705)
Supplement: Supplementary file 1 [file ijms-24-13705-s001.zip › ijms-2560880-supplementary.pdf]

Supplementary Materials

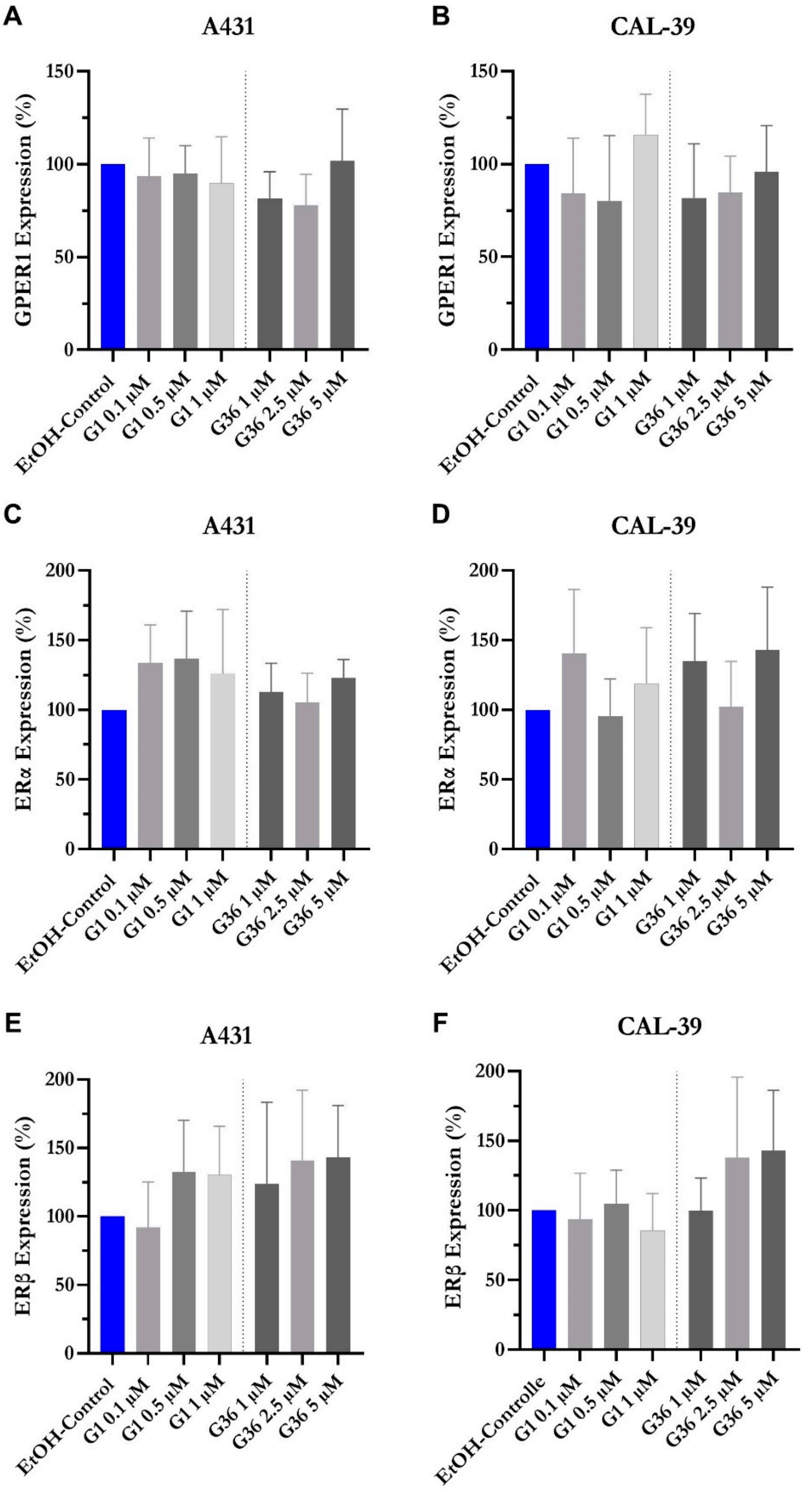

Figure S1. Effects of GPER1 agonist G1 and antagonist G36 on estrogen receptor expression in vulvar carcinoma cells. Expression of GPER1 (**A**,**B**), ERα (**C**,**D**), and ERβ (**E**,**F**) after treatment with G1 or G36 on vulvar carcinoma cells A431 (**A**,**C**,**E**) and CAL-39 (**B**,**D**,**F**). n = 3 (**B**), n = 4 (**A**,**C**–**F**).

Table S1. Densiometric evaluation of the effects of GPER1 agonist G1 and antagonist G36 on expression of GPER1 (**A**,**B**), ERα (**C**,**D**), and ERβ (**E**,**F**) in A431 (**A**–**E**) and CAL-39 (**B**–**F**) vulvar carcinoma cells. Mean ± SEM % of control (= 100%).

(**A**)

Treatment	Mean (%)	SEM	*n*
Ethanol control	100.00	0.00	4
G1 0.1 µM	93.40	20.84	4
G1 0.5 µM	95.05	15.07	4
G1 1 µM	89.73	25.12	4
G36 1 µM	81.59	14.29	4
G36 2.5 µM	78.12	16.47	4
G36 5 µM	101.99	27.68	4

(**B**)

Treatment	Mean	SEM	*n*
Ethanol control	100.00	0.00	3
G1 0.1 µM	84.27	29.72	3
G1 0.5 µM	80.24	35.03	3
G1 1 µM	115.76	21.87	3
G36 1 µM	81.76	29.17	3
G36 2.5 µM	84.69	19.65	3
G36 5 µM	95.96	24.69	3

(**C**)

Treatment	Mean	SEM	*n*
Ethanol control	100.00	0.00	4
G1 0.1 µM	133.64	27.28	4
G1 0.5 µM	136.70	34.18	4
G1 1 µM	126.08	46.03	4
G36 1 µM	112.98	20.42	4
G36 2.5 µM	105.37	21.02	4
G36 5 µM	123.06	13.05	4

(**D**)

Treatment	Mean	SEM	*n*
Ethanol control	100.00	0.00	4
G1 0.1 µM	140.81	45.68	4
G1 0.5 µM	95.72	26.48	4
G1 1 µM	119.13	39.77	4
G36 1 µM	135.00	34.24	4
G36 2.5 µM	102.47	32.32	4
G36 5 µM	142.84	45.29	4

(**E**)

Treatment	Mean	SEM	*n*
Ethanol control	100.00	0.00	4
G1 0.1 µM	92.03	33.18	4
G1 0.5 µM	132.60	37.71	4
G1 1 µM	130.47	35.42	4
G36 1 µM	123.89	59.57	4
G36 2.5 µM	141.02	51.20	4
G36 5 µM	143.29	37.72	4

(**F**)

Treatment	Mean	SEM	*n*
Ethanol control	100.00	0.00	4
G1 0.1 µM	93.75	33.04	4
G1 0.5 µM	104.93	23.91	4
G1 1 µM	85.57	26.59	4
G36 1 µM	99.69	23.64	4
G36 2.5 µM	137.88	57.89	4
G36 5 µM	142.85	43.33	4
